# Transcranial direct current stimulation as a motor neurorehabilitation tool: an empirical review

**DOI:** 10.1186/s12938-017-0361-8

**Published:** 2017-08-18

**Authors:** Ana Sánchez-Kuhn, Cristian Pérez-Fernández, Rosa Cánovas, Pilar Flores, Fernando Sánchez-Santed

**Affiliations:** 1 University of Almería, Almería, Spain; 2 Instituto de Neurorehabilitación Infantil InPaula, Almería, Spain

## Abstract

The present review collects the most relevant empirical evidence available in the literature until date regarding the effects of transcranial direct current stimulation (tDCS) on the human motor function. tDCS in a non-invasive neurostimulation technique that delivers a weak current through the brain scalp altering the cortical excitability on the target brain area. The electrical current modulates the resting membrane potential of a variety of neuronal population (as pyramidal and gabaergic neurons); raising or dropping the firing rate up or down, depending on the nature of the electrode and the applied intensity. These local changes additionally have shown long-lasting effects, evidenced by its promotion of the brain-derived neurotrophic factor. Due to its easy and safe application and its neuromodulatory effects, tDCS has attracted a big attention in the motor neurorehabilitation field among the last years. Therefore, the present manuscript updates the knowledge available about the main concept of tDCS, its practical use, safety considerations, and its underlying mechanisms of action. Moreover, we will focus on the empirical data obtained by studies regarding the application of tDCS on the motor function of healthy and clinical population, comprising motor deficiencies of a variety of pathologies as Parkinson’s disease, stroke, multiple sclerosis and cerebral palsy, among others. Finally, we will discuss the main current issues and future directions of tDCS as a motor neurorehabilitation tool.

**Electronic supplementary material:**

The online version of this article (doi:10.1186/s12938-017-0361-8) contains supplementary material, which is available to authorized users.

## Background

The central nervous system (CNS) works thanks to the communication between more than 100,000 millions of neurons, whose activity and networking is modulated by chemical and electrical processes [1]. Across history, humans have been trying to alter the electrical brain processes to enhance human’s brain function, for the treatment of psychopathologies and for a better understanding of the brain physiology. For example, in the antiquity, modulation of the electrical processes of the brain started with the use of electrical impulses of torpedo fishes applied directly on the CNS, for therapeutic purposes [2]. In 1746, Musschenbroek (1692–1761) used Leyde jars and electrostatic devices to treat neuralgia, contractures and paralysis. The discovery of biometallic electricity and the invention of the voltaic battery augmented the interest in the therapeutic effects of galvanism. Afterwards, Duchenne de Boulogne (1806–1875) upgraded the electrotherapy with volta and magnetofaradaic apparatuses. Fortunately, in the past Century, the technological advances and its integration in health sciences have let us go from uncontrolled and unsafe interventions with side effects to well-controlled, more effective and safe stimulation devices [3].

Currently, the most used stimulation devices can be divided into invasive techniques, such as deep brain stimulation (DBS), and non-invasive brain stimulation (NiBS) techniques, whose most representative methods are transcranial magnetic stimulation (TMS) and transcranial direct current stimulation (tDCS) [4].

Although results are variable [5], DBS has reported positive results over the motor function, especially on the motor symptoms of Parkinson’s disease. However, DBS is a technique that needs the implantation of the electrodes on the stimulated area, which is associated with the typical risk derived from surgery, as infections. Therefore, there is an increasing tendence on the search for non-invasive brain stimulation techniques, which can modulate the motor function avoiding those risks.

Hence, NiBS are characterized for its easy and safe use and relatively cheap price, demonstrating also successful results in the treatment of neurological and psychiatric alterations [4]. In the last decades, TMS has been the most researched and developed neuromodulation technique. TMS generates fast changes in the magnetic field delivering electrical currents through the brain, allowing the specific modulation of the cortical excitability through the initiation of action potentials [6]. Multiple studies have already shown its efficacy and safe use for the treatment of multiple pathologies [7], serving also as a useful tool for the functional location of brain areas, especially regarding the motor cortex [8, 9]. However, TMS requires the participation of the participant, and due to its functioning, it is difficult to perform a sham condition, which is highly desirable especially in the research field. In addition, TMS produces in most of the cases undesirable side-effects, as headache [10].

Therefore, the tDCS technique is attracting a strong interest in the neuroscience research field. tDCS has supposed a revolution in the last 15 years of research, solving most of the disadvantages of TMS [10]. tDCS is a neuromodulation tool consisting on a battery connected to two electrodes, the anode and cathode, which are placed directly over the brain scalp and over extracephalic regions. The current flows between both electrodes and induces the depolarization or hyperpolarization of the membrane of the underlying neurons, which depends of the anodal or cathodal nature of the electrode [11], altering the neuronal excitability resulting in the modification of the brain activity [12]. This device is completely portable, as it is provided by built-in rechargeable battery with duration of approximately 6 h stimulation time at 1 mA (0.5–1.5 W of power consumption), and needs approximately 7 h for complete recharging. In addition, including battery, it has a weight of 0.8 kg. Its portability is one of the biggest advantages of tDCS in the context of NiBS. Therefore, tDCS can be considered as a suitable complementary technique on motor rehabilitation therapy, allowing its application in different contexes, during the motor training and even combined with aerobic exercise [13, 14].

This non-invasive brain manipulation has opened the doors for a variety of potential treatments for the major neurological and psychiatry diseases [15], as depression [16], schizophrenia [17], Obsessive–Compulsive disorder [18] and addictions [19], among others.

However, motor functions are the major target for clinical and non-clinical studies regarding tDCS, serving mainly as a potential tool in post-stroke rehabilitation [20], but also in pathologies like Parkinson’s disease [21]. In addition, numerous studies have shown that tDCS produces changes in the brain plasticity processes, generating long-lasting effects that enhances even further its applicability in the neurorehabilitation field [22, 23].

The purpose of this review is to assess the current and future stage of tDCS regarding its use on the human motor function, identifying the empirical cues that point out its benefits as well as its potential limitation, providing a comprehensive framework for designing future research in the field of brain stimulation with tDCS and human motor rehabilitation. The present review is divided in four parts. The first part is based on a detailed definition on what we know about tDCS, the protocols of montage and parameters of stimulation, comprising the mechanisms of action of tDCS, what differs tDCS from other non-invasive neuromodulation techniques, and the main need to-know safety standards. Given the conciseness of this first part, we will present the recent studies focusing exclusively on the empirical data obtained from the use of tDCS in the human motor function, regarding, in the second part, healthy humans; in the third part, its clinical application on deteriorated human motor functions across different pathologies as Parkinson disease, stroke and cerebral palsy. Finally, in the fourth part of this review, we will discuss the main current issues of tDCS applied on the human motor function.

## Understanding and differentiating tDCS

### Definition of tDCS

Transcranial direct current stimulation is a non-invasive neurostimulation technique that delivers a weak direct current towards the cortical areas situated under two opposite electrodes: depending on the aim of the study, the “active electrode” is the one located on the target area, and the other one is the “reference electrode”. These electrodes are connected to a 9-volt battery and covered by conductive sponges soaked in saline or other conductive media [24, 25]. When both electrodes are located over a conductive area, as the scalp, a direct current flows between the anode and the cathode, generating specific changes in the cortical excitability of the underlying tissue [26]. These changes can be manifested into behavioral changes and even neuroplasticity might be generated under the use of tDCS through long-term potentiation (LTP)-like processes [27]. Therefore, this technique has attracted a great attention in the past century in the area of neuroscience research and neurorehabilitation.

### Effective stimulation parameters and montage protocol

One of the key points in the practical use of tDCS is the choice of the correct stimulation parameters. For a safe and effective use of tDCS, it is important to delimit the current intensity, which is generally expressed in milliamps—mA, but also in amps—A and/or microamps—µA. In research, the current intensity is selected normally in a range from 0.5 to 2 mA [12].

The other most important stimulation parameter is the size of the electrodes, which varies between 4 × 4 (16 cm^2^), 5 × 5 (25 cm^2^), 5 × 7 (35 cm^2^) and 6 × 6 (36 cm^2^) [24]. However, the current intensity and the electrode size are two interrelated parameters, which lead to the current density parameter. The current density is the result of the current intensity divided by the total surface volume that the electrode occupies, and it is expressed in research as µA/cm^2^, mA/cm^2^ and A/cm^2^ [28], being the most used range between 0.028 and 0.06 mA/cm^2^ [28]. Smaller electrodes might be more efficient [24]. For instance, in a study that compared three electrode sizes (12 m^2^, 34 cm^2^ and 35 cm^2^) while maintaining the same current density, the anodal tDCS with a 12 cm^2^ active electrode showed a greater spatial focality of the stimulated area, and evoked larger corticospinal changes, expressed by higher motor evoked potentials (MEPs) [29].

The location of the active electrode is generally determined by the international electroencephalogram (EEG) 10-20 System [30], and it is generally placed on the area that represents the motor cortex (C3–C4). In the major studies regarding motor regions, the reference electrode has been placed over the contralateral orbit (just above the contralateral eye) [21], but it is also usually placed on extracephalic regions (neck, arms, chin, etc.) [12]. Recent studies, especially focused on stroke patients, have determined that bihemispheric stimulation, with the active electrode on the damaged hemisphere, and the reference electrode on the healthy hemisphere, could contribute to the reduction of the stroke motor symptoms, probably due to the reestablishment of the abnormal interhemispheric inhibitory interactions after stroke, reducing the increased activation of intact ipsilesional primary and adjacent nonprimary motor regions [31].

tDCS has its largest effects under the electrodes [32], however, studies with functional magnetic resonance (fMRI) and positron emission topography (PET) have showed that the stimulation generates constant and generalized effects in other areas of the CNS [33]. EEG results point in the same direction [34]. That means that the stimulation of one area will probably affect other brain areas throughout neural circuit networks [35]. This is not surprising due to the complex anatomy and interconnectivity of the human CNS, but it evidences the necessity of an exhaustive analysis of the stimulated structure and its relationship with adjacent areas [36, 37].

### What differs tDCS from other non-invasive neurostimulation techniques

Deep brain stimulation is a neurostimulation technique that works thanks to the implantation of electrodes in the precise target brain locations, showing until now suitable results for the treatment of motor [38]. However, tDCS differs from DBS for the fact that it does not need surgery for its application. Therefore, the present technique is able to stimulate the brain in a non-invasive way, which reduces risks and possible side effects. Nevertheless, DBS is able to reach deep brain areas, while tDCS reaches only cortical areas [12].

As mentioned before, TMS is also a well-studied NiBS technique that has shown numerous successful results in the area of neurostimulation in healthy, as well as in clinical studies. However, TMS frequently produces headache in most of the users [39], whereas tDCS can provoke itchiness in the location of the electrodes and not greater side effects have been demonstrated until now [12, 40]. In addition, TMS is a relative noisy device, whereas tDCS is a portable, totally silent and discrete device that does not need the collaboration of the user, which facilitates the creation of a *sham* condition, and consequently, its usage in the research field. This *sham* condition is generally performed with real stimulation, but only the first minute, and subject do not perceived it as different from the stimulation condition [41]. As we will see in the following section, the mechanisms of action of tCDS are different than those of other neuromodulation technique, as, in contrast with TMS, tDCS does not induce directly neuronal action potentials, because static fields in this range do not profit the fast depolarization needed to produce action potentials in neural membranes [12]. Consequently, tDCS should always be administered together with motor training tasks, showing its highest effects when applied before or during the task [42]. A recent study also has compared the electric field distribution in TMS and tDCS in the motor cortex showing that both techniques induce electric fields, but with different directions: the field in tDCS is mainly perpendicular to the cortical surface, while in TMS the field is regularly parallel to it [43].

### Mechanism of action of tCDS

Despite the exact mechanisms of action of tDCS are still under study, it is widely established that the current flows between the anode and the cathode produce a modulation of the resting membrane potential of the underlying neurons of the stimulated area. In the case of the anode, the neuronal excitability generally increases and the cell membranes are depolarized, while in the area of the cathode, the excitability is dropped down and therefore producing a hyperpolarization of the underlying tissue. However, it is important to highlight that, depending on the direction and the current intensity, this effects can be inverted [12]. After the stimulation, the MEPs levels produced by a single 10 min session can last until 60 min after the stimulation [44].

Short as well as long lasting effects produced after the application of tDCS are important in order to differentiate its effects: short term effects are interesting in the research field, while long term effects are necessary to be considered as part of a treatment in the clinical field. Short-term effects seem to be produced by local changes in the ionic concentrations, specific alterations in transmembrane proteins and electrolysis related to slight changes in the hydrogen protons concentration [34]. Actually, recent studies have observed a large accumulation of myoinositol into the phospholipid membrane after anodal tDCS through proton magnetic resonance spectroscopy [45] and a significant augmentation of oxyhemoglobin concentrations after 1 mA of anodal tDCS by near-infrared spectography [46], showing also changes in the cerebral blood flow when applied over the primary motor cortex [47].

The electrical current produced by tDCS modulates the resting membrane potential of a variety of neuronal population, as pyramidal cells, and interneurons (gabaergic). This leads to substantial changes in the field potential; and these changes raise or drop the firing rate up or down, depending on the nature of the electrode [48].

In consonance with this, several studies have showed sustained neurochemical differences between both types of stimulation, anodal and cathodal. Many investigations are in agreement with the fact that the excitatory effects of tDCS are mediated, at least in part, by an important reduction of the GABAergic activity and by the facilitation of the NMDA glutamatergic receptors [49]. On the other hand, it seems that cathodal tDCS is mediated by an important reduction of the excitation of the glutamatergic system [50].

The key point of tDCS to be considered as a tool in neurorehabilitation is the possibility of producing long-term effects. These long-term effects are mediated by the neuroplasticity generated by the application of tDCS, which has been confirmed in a variety of animal and human studies [51–53]. The consolidation of learning during the use of tDCS is possibly mediated by catecholaminergic transmitters, and specially adrenergic ones, being the catecholaminergic effects NMDA receptor-dependent [54]. The positive effects produced by the modulation of the glutamatergic system could eventually lead to the release of the brain-derived neurotrophic factor (BDNF) [55]. In fact, tDCS has shown to change the BDNF [56] promoting the BDNF-dependent synaptic plasticity [22].

### Safety considerations before using tDCS

The main facts that determine the safe and correct use of this technique are the current density, the duration of the stimulation, the number of sessions and the inter-session time [26, 57].

Considering that the high current density is concentrated in the edges of the electrode, special attention needs to be payed on the intensity and characteristics of the electrodes; actually, some studies have developed circular electrodes instead of square-shaped ones. This has to be added to the importance of the control of the saline quantity incorporated to both sponges, which facilitates the electric current transmission [58].

Indirect biomarkers of brain damage did not change after the application of tDCS: the serum levels of the molecular markers of neuronal lesion (N-acetylasperate) as well as the levels of other related metabolite contents remained stable after the application of tDCS [45]. Also no adverse effects were found on the heart function [59] and no convulsive effects have been related to tDCS [40].

In a safety study, 1 mA of tDCS was applied across 567 sessions on 102 healthy participants, and no relevant adverse effects were seen, showing tDCS better side effects than previous studies with TMS. tDCS seems to be a safe and suitable-for-use methodology that produces side effects only in punctual occasions [60]. Some side effects that can commonly been observed are soft itching, mild burning or slight pain felt under the electrodes. In addition, subtle tingle and moderate fatigue. Less common, it is possible to experiment slight headache, difficulty to concentration, nausea and sleep disturbances [60]. Depending on the electrode shape and size, infrequently, tDCS can produce a skin-burning lesion [61]. However, the skin lesion would have any consequence for the cerebral tissue [62]. For the correct electrical conduction between the electrodes and the skin, saline has demonstrated to be safer than tap water. If electrode gel is used, the quantity has to be large enough to avoid the direct contact between the electrodes and the skin. However, the most important issue to avoid skin damage is to ensure that the skin surface is not dry [63].

## Effect of tDCS on the motor function in healthy population

This second part of the review will focus on the most prominent effect found on the application of tDCS on the human motor function on healthy participants. These outcomes are the most promising steps forward to reach a limited and effective use of tDCS on the neurorehabilitation field. The principal key facts of the studies exposed in this section can be found in Additional file 1: Table S1.

### Effect of tDCS on lower limbs

The stimulation on the primary motor cortex (M1), when applied over the contralateral hemisphere of the target leg has reported positive results. For instance, in the study of Sriraman et al. [13], 15 min of 1 mA of anodal tDCS showed better results 24 h after the stimulation than the sham condition. In addition, when the stimulation was applied together with an ankle motor task, the results obtained where even better. These results were measured by the accuracy showed on a manipulandum for ankle motor testing and practice, were the participants performed an ankle dorsiflexion and plantarflexion to match a sinusoidal wave on the computer screen as accurately as possible.

Moreover, in a study where the intensity was configured up to 2 mA, the anodal stimulation of the M1 contralateral to the target leg during 10 min, reported an increased positive work generation during propulsion, step length and slow walking speed, enhancements that lasted until 45 min after the stimulation. In the same study, bicephalic and monocephalic stimulation configuration where compared, showing bicephalic configuration stronger effects than the monocephalic one. However, large inter-individual variability was observed [64].

But when concerning human motor modulation, not only the motor cortex is a stimulation target, also the stimulation of the cerebellum is a potential target area for the motor rehabilitation. Galea et al. [65], applied anodal tDCS (atDCS) and cathodal tDCS (ctDCS) on the right cerebellum cortex during 25 min, and obtained a tone inhibition after cathodal stimulation and an increase after the anodal stimulation. In addition ctDCS decreased the cerebellum-brain inhibition (CBI) action, and augmented after atDCS.

For instance, Jayaram et al. [66] applied anodal and cathodal tDCS on both cerebellum hemispheres (3 cm lateral to the inion) during 15 min with a current intensity of 2 mA. atDCS increased the rate of walking adaptation, while ctDCS had the opposite effect. In the previously mentioned study, atDCS was recognized as a CBI depressant, which is contradictory. Therefore, additional research is needed in order to clarify this paradoxical effect (for possible hypothesis, see Jayaram et al. [66]).

Neuroimaging studies give us the possibility to confirm the changes in the brain beyond the behavioral improvements. In fact, in a study that stimulated with atDCS the left M1 with 2 mA, during 15 min, across 4 days [67], fMRI showed an activation of the right supplementary motor area and a decrease of the activation of the contralateral hemisphere. But also a bilateral activation of diverse structures such as the anterior cingulate gyrus, the right middle/superior temporal gyrus, middle/superior frontal gyrus and the primary and secondary somatosensory cortices were detected. These results indicate that atDCS changes the excitability of the corticospinal pathway to both legs by networks, which enroll many areas besides M1.

Added to this, MEPs and muscular outcomes by electromyography (EMG) have been studied in healthy humans. Thus, Madhavan and Stinear [68] have demonstrated that, after the application of atDCS on both M1 (leg representation), but in separated sessions and with a current intensity of 0.5 mA (0.06 mA/cm^2^) during 10 min, strong inter-variability data measured in 40 muscle pairs (10 subjects × 2 sessions × 2 muscle pairs) was observed. Instead, a mean change in cortical excitability of 60% on stimulated areas was also found, but induced between-hemisphere opposite sign modulation, as authors previously hypothesized, disturbed most part of physiological outcomes. This is a clear proof of the complexity of human cortex. Instead, we consider that a higher current intensity could be needed in order to induce strongest contralateral modulation in both MEPs and EMGs, despite of the fact that similar current density was demonstrated as enough to induce consistent MEPs changes by Jeffery et al. [69]. These authors implemented a current intensity of 2 mA during 10 min on the M1 (leg representation), with an increase in MEPs amplitude of 59% at rest and 35% in contraction phase, with effects further to 60 min after stimulation. All these data taken together indicate that, even being the current density value very important, every single feature of the stimulation procedure has to be studied and planned in depth.

### Effect of tDCS on upper limbs

In order to predict the effects of tDCS on the primary motor cortex on an injured or under-used motor function, an experimental design widely used on healthy participants is the stimulation of the contralateral motor cortex of the non-dominant hand. The under-use of the left hand, by right-handed participants or the right hand by left-handed participants represents an effective model to study the effect of training complemented by tDCS on motor learning [70].

An example of the success of this kind of experimental design, is the study carried on in our laboratory (Sánchez-Kuhn et al. unpublished) in which a constant current of anodal 2 mA tDCS was administered during 20 min on the right motor cortex within three consecutive sessions, with an intersession time of 24 h, during the training with the non-dominant hand on the sequential finger tapping task (SEQTAP). Participants were tested before the experiment, during the three training sessions, in the short term (20 min after), and in the long-term (8 days after). Findings showed a higher performance of the stimulated participants when compared to the sham group during the performance of the task, in the short-term, and also in the long-term. Other studies have also successfully improved the finger movement on healthy individuals by anodal tDCS [71].

The voluntary movement of responding to a stimulus can be modified, but also the motor suppression has been improved with anodal tDCS [72] which lets the doors open for the research of possible treatments for the compulsive behaviors or stereotypes manifested in the Obsessive–Compulsive disorder or in autism, among others.

## Effect of tDCS on the motor function in clinical population

The following section will focus on the most remarkable effects found on the application of tDCS on the human motor function of clinical population. The reviewed pathologies have all in common the deterioration of the motor skills as a symptom or consequence of the disease. The principal key facts of the studies exposed in this section can be found in Additional file 2: Table S2.

### Stroke and tDCS

Dysfunctions in the use of upper limbs and deficits in the functional walking are among the most common after stroke. Only 5% of adult post-stroke patients recover their full upper limb function [73]. Motor recovery after stroke is mostly dedicated to the intent to preserve the ipsilesional motor networks and the interactions between both hemispheres. tDCS seems to be able to modulate these processes [74]. Following this theory, it has been demonstrated that positive effects can be observed by the cathodal stimulation of the non-affected hemisphere, presumably due to the current flows property thanks to the undamaged intracortical networks [75].

In accordance with this approach, Lindenberg et al. [31] combined bicephalic stimulation (atDCS on ipsilateral M1 and ctDCS on contralesional M1) (1.5 mA/30 min/5 sessions) with orthodox occupancy and physical therapy, obtaining an enhancement of the motor functions of chronic post-stroke patients when comparing with the sham condition. However, contrary data to this approach has been found. For instance, other study performed on healthy subjects showed that a single session of only atDCS or ctDCS (1 mA/20 min) displayed greater effects over the level of MEPs than bicephalic stimulation [76].

In another way, a significant enhancement assessed in the Jebsen-Taylor hand function test (a specific hand function task for daily living activities) was observed after the application of atDCS on M1 (1 mA/20 min) on post-stroke patients with hemiparesis subsequent to first-ever unilateral stroke, but not on the sham condition, resulting in functional improvements in motor function of the paretic hand [77].

Similar unilateral tDCS effects have been also confirmed also on further functions deteriorated after stroke. For example, post-stroke aphasia has been chose as a key objective for tDCS intervention. Rosso et al. [78] demonstrated, after the application of ctDCS over the right Broca’s area, an improvement in language performance in patients with aphasia in the chronic post-stroke phase, supporting the idea that ctDCS can suppress inhibitory inter-hemispheric influences from the right Broca’s area to the affected one, and that inter-individual differences are crucial in the design of the methodology of effective stimulation processes. Those effects might be facilitated by GABAergic intracortical and inter-hemispheric function [79].

### Dysphagia and tDCS

One of the possible motor dysfunctions derived from stroke is dysphagia, which is a high disrupting syndrome that consists basically on the impossibility of starting and accomplishing the voluntary or involuntary movement of swallowing. Dysphagia might be followed by serious consequences as nutritional problems, respiratory system issues and daily life deficits affecting the emotional and social areas of the patient [80, 81].

The primary motor cortex plays the principal role on the voluntary initiation of the swallowing process, and both hemispheres seem to be responsible for this behavior [82]. However, neuroimaging techniques showed that in most of the cases, the activation during the swallowing process was greater in one hemisphere than in the other, being this fact independent of handedness. Furthermore, this predominance was different between identical right-handed twins [83].

In the last years, studies have been developing the possibility of incorporate tDCS to the treatment of dysphagia (for review, see Sandrini and Cohen [84]). Kumar et al. [85] applied atDCS on the undamaged motor cortex (2 mA/30 min/5 sessions) together with standardized swallowing training, finding a significant improvement of the Dysphagia Outcome and Severity Scale (DOSS) scores. Also Yang et al. [86] used atDCS (1 mA/20 min/10 sessions) but on the affected hemisphere, accurately, on the over the pharyngeal motor cortex, and measured the effects of atDCS by the functional dysphagia scale (FDS) using video fluoroscopic swallowing measure (VFSS) immediately after the intervention and three months later. Differences between the atDCS and the sham group emerged on the second evaluation.

In our laboratory (Sánchez-Kuhn et al. unpublished), we carried on a study over a post-stroke cellebelar lesion patient who consequently developed dysphagia. This study combined the monocephalic 1 mA anodal tDCS stimulation during 16 sessions of 20 with swallowing training. However, only minor positive results were registered over dysphagia symptoms as well as over The swallowing quality of life questionnaire (SWAL-QoL). Nevertheless, diffusion tensor imaging (DTI) results showed a significant increase of the number of fibers and connections in the left cerebellum after the combined treatment of anodal tDCS over M1 and swallowing training. Therefore, tDCS delivers also information about the brain networks involving motor functions as the swallowing process.

### Parkinson’s disease and tDCS

Besides DBS has shown positive results on the treatment of Parkinson’s disease (PD) (for review, see Hickey et al. [87]), many studies have reported positive results of tDCS on motor as well as cognitive symptoms of Parkinson’s disease, data that is supported also by neurophysiological effects (for review, see Broeder et al. [88]). However, in the current section, we will focus on the particular effects of tDCS on motor deficits. Therefore, Fregni et al. [89] demonstrated that, with the application of atDCS (1 mA/20 min/1 session) on the left M1 of patients in OFF-state, a significant increase in MEPs were produced compared to sham condition. This effect was contrary after ctDCS, as ctDCS decreased the MEPs amplitude. These effects correlated also with motor enhancements (bradykinesia, tremor, rigidity, gait, postural instability etc.). Augmenting the number of sessions up to 5, a study found also positive results after atDCS (2 mA/20 min) but in patients in ON-state (phase when Parkinson’s motor symptoms are generally under control) [90].

Recently, a study that induced dyskinesias (involuntary muscle movements that characterize the motor symptoms of Parkinson) with Levodopa in PD patients, found a reduction of the dyskinesias when administering bilateral atDCS (2 mA/20 min/5 sessions) in M1 as well as in the cerebellum [91].

In addition, tDCS has resulted in positive motor outcomes on the long-term, as seen in the study of Benninger et al. [21] where the group that received the stimulation improved bradykinesia in their upper extremities in both, the on and the off state, effect that was maintained for longer than 3 months.

### Multiple sclerosis/Amyotrophic lateral sclerosis and tDCS

Few studies from 2010 have focused on different motor and cognitive effects of the application of tDCS in patients with multiple sclerosis (for review, see Mehta et al. [92]; Pérez-Fernández et al. [93]). Thus, Cuypers et al. [94] demonstrated that, after the application of atDCS with a current intensity of 1 mA during 20 min on M1 (first dorsal interosseous), contralateral to the more impaired hand, significant corticospinal excitability increase was observed evaluated by MEP variations, effect non-observed after sham stimulation. This cortical modulation triggered to a recruitment-curve plateau increase, something that could be explained by distal effects mediated by large-diameter myelinated axons. Nevertheless, no functional effects were studied. However, no motor improvement facilitation were observed by Meesen et al. [95] after atDCS with a current intensity of 1 mA during 20 min on contralateral to impaired hand M1, compared to sham condition. Further researches in motor function are needed.

It is interesting to point out that also positive sensory modulations have been observed after atDCS application in patients with multiple sclerosis. Mori et al. [96] demonstrated that, by applying atDCS with a current intensity of 2 mA, during 20 min/5 consecutive daily stimulation on somatosensory cortex (S1), temporally ameliorated sensory deficits (spatial discrimination thresholds on the hypoesthetic hand) further to 2 weeks after treatment were observed in patients with multiple sclerosis. These sorts of positive sensory modulations have been observed also in pain self-sensation in patients with multiple sclerosis [97], after the application of atDCS with a current intensity of 2 mA, during 20 min/5 consecutive daily stimulation on contralateral to somatic painful area M1, with a clear decrease of values in standardized pain scales.

On the other hand, there are very few studies of the application of tDCS on Amyotrophic lateral sclerosis (ALS). However, as Di Lazzaro et al. [98] pointed out, after the variable results of the application of TMS in patients with ALS, tDCS could be considered as a better intervention tool due to its longer-lasting effects on cortical excitability. Thus, and as a preliminary study, these authors showed no significant effects after ctDCS on M1 (the cortical representation of the first dorsal interosseous muscle), with a current intensity of 1 mA during 20 min on both hemispheres in two different patients. Related to this, Munneke et al. [99] demonstrated no significant cortical excitability variations after 1 mA ctDCS during 7, 11 and 15 min. However, an important effect was observed in healthy subjects, indicating that patients with ALS could have less responsive corticospinal pathways to the inhibitory ctDCS effects. Such results were early demonstrated by Quartarone et al. [100] in both anodal and cathodal stimulation types. Nevertheless, it has been demonstrated that continuous theta burst stimulation (cTBS) by TMS can induce an inhibitory effect on corticospinal excitability in patients with ALS only after five daily sessions [101], so it is reasonable to postulate that repetitive ctDCS training could generate similar effects on patients with ALS.

### Spinal cord injury and tDCS

After the numerous confirmations of the positive effects of tDCS and TMS on the management of neuropathic pain after spinal cord injury [92], the last investigations point towards tDCS as a target tool in the treatment for the motor-related consequences of spinal cord injury. Thus, Silva et al. [102] applied atDCS on both M1 (2 mA/12 min) in a subject with total chronic spinal cord injury and the results showed a general improvement in exercise tolerance by the specific measures undertaken in exercise time and power, perceived exertion, glucose levels, and the time needed to reach the heart rate threshold.

In the study of Murray et al. [103], after the application of atDCS on the left M1 (extensor carpi radialis muscle representation) in nine patients with chronic spinal cord injury (2 mA/20 min/3 sessions), the authors observed an increase of 40% in the corticospinal excitability (MEPs) amplitude. This result was not reached with an intensity of 1 mA. Despite of the high current density implemented in this work, no significant adverse effects were seen.

However, the best results in spinal cord injury were obtained applying directly the new technique of transcutaneous spinal direct current stimulation (tsDCS). Hence, Hubli et al. [104] located the active electrode longitudinally between the spinous processes T11 and T12 (2.5 mA/20 min). This intervention showed specific differences in spinal reflex behavior, where patients showed higher changes in spinal reflex amplitude after a-tsDCS than healthy subjects, exhibiting even better results than receiving a session of assisted walking in the driven gait orthosis “Lokomat”. These results are evidenced by the changes that produced atsDCS in the conduction along the lemniscal pathway (specific somatosensory evoked potentials amplitude P30) in healthy subjects after an application of atsDCS over the spinous process (T10) (2.5 mA/15 min) [105].

### Restless legs syndrome and tDCS

The restless legs syndrome (RLS) is a sensorimotor neurological pathology whose main characteristic is periodic limb movements during sleep, which generates alterations in sleep [106]. This alteration has been insufficiently studied in tDCS approaches, and controversial data has emerged. However, Heide et al. [107] found that after the application of both atDCS and ctDCS over the spinal process T1 with a current intensity of 2.5 mA during 15 min in patients with RLS, an important decrease after anodal stimulation in spinal excitability was observed, conducting to clear reduction of restless symptoms in a VAS scale and in specific reflexes. Such effects were not observed after sham stimulation.

Nevertheless, such effects have not been observed after cortical stimulation [108]. Those authors implemented tDCS on bilateral M1 (CZ position, legs representation) with a current intensity of 2 mA during 20 min for 5 sessions/2 weeks. No significant differences were found between groups in the International RLS Group Rating Scale and the Clinical Global Impressions-Improvement. All these data together could indicate that, in this sort of pathologies, direct currents on the back could be a better rehabilitation procedure than cortex stimulation approach. However, further researches are needed.

### Cerebral palsy in children and tDCS

Cerebral palsy is the most common motor disease in children, and it refers to permanent, motor development disorders owing to a primary brain lesion, causing secondary musculoskeletal problems and subsequently, constraining the daily living activities of the child [109].

Besides there are numerous studies reporting the safe application of tDCS on infancy special actions are needed when administering tDCS on children, as the current intensity, the density and the size of the electrodes (for review, see [110]).

As in the previous studies, the effects of tDCS on cerebral palsy are especially positive when the stimulation is administered in combination with motor rehabilitation. On a study with 24 children with cerebral palsy, the researches administered atDCS (1 mA/20 min/10 sessions) combined with treadmill training on balance and functional performance. The Pediatric Balance Scale and the Pediatric Evaluation of Disability Inventory results showed positive effects on the balance score of the stimulated group, 1 and 4 weeks after the treatment [111].

Spasticity has been also a key target in the use of tDCS on children with cerebral palsy, as it is one of its most common symptoms [112]. Spasticity is defined as an upper motor neuron syndrome characterized by a velocity-dependent increase in the tonic stretch reflexes with amplified tendon jerks resulting from the hyperexcitability of this reflex [112]. Concerning the importance of this symptom in cerebral palsy, Aree-Uea et al. [113] conducted a study with 46 children between 8 and 18 years with cerebral palsy. The treatment consisted of a stimulation of atDCS over the left primary motor cortex (1 mA/20 min/5 sessions) added to stretching exercises during five consecutive sessions. Spasticity was measured before and 24 and 48 h after the treatment. Results showed a reduction of finger spasticity immediately after the treatment, a reduction of the elbow spasticity also immediately and 24 after the treatment and a reduction of wrist spasticity immediately, 24 and 48 h after the treatment with atDCS.

Moreover, a number of promising studies is combining tDCS with virtual reality training, presenting optimistic results in researches concerning motor abilities, as for example, on the improvement of the body sway velocity [114], and also regarding spatiotemporal gait variables (velocity and cadence) and gross motor function and mobility [115]. Furthermore, in this last study anodal tDCS led to a significant change in motor cortex plasticity, as evidenced by the increase in the amplitude of the motor evoked potential. The experiential learning facilitated by virtual reality training, where the stimulus are visual, and provide feedback, augment the motivation of the children, which can lead in even higher positive effects of the stimulation with tDCS (for review, see [116]). Therefore, the combination of motor training with virtual reality and tDCS could constitute a new potential neurorehabilitation therapy for children with cerebral palsy.

## Limitations and future guidelines

Even though the extend literature available about tDCS, there are still several limitations that have to be taken into account. As mentioned before, the parameters of stimulation must be exactly defined in order to reach the desired effects. This is not an easy task concerning that not always higher intensity and a longer stimulation means larger effects of tDCS. In addition, the measure of the improvements should be compared with the results in other tasks, to ensure the specific motor, behavioral or cognitive effects of the stimulation. Furthermore, to consider tDCS as a neurorehabilitation tool, it is essential to find a long-lasting effect of tDCS that ensures improvements not only over hours or days, but also month and even years (for reviewing this and other tDCS limitations, [117]).

However, one of the most important issues concerning the effective use of tDCS is the interindividual variability found across the data until now. The effects of tDCS are brain state-dependent [63], consequently, the amount and direction of its effects is critically depending on the previous physiological state of the target neural structures [63, 118]. Wiethoff et al. [119] found a large variability regarding corticospinal excitability response to tDCS across 53 healthy subjects. According to the most recent studies regarding the interindividual variability there are some different hypothesis why tDCS might produce different effects. Up to date, the tendence of the results show that those participants with lower baseline performance result more benefitted from tDCS. Moreover, in our laboratory (Sánchez-Kuhn et al. unpublished), we conducted a study including healthy participants with and without previous musical training, who performed a SEQTAP task with the non-dominant hand while being stimulated over the contralateral primary motor cortex. Among healthy subjects, musicians offer an outstanding human model for studying brain properties of acquiring, practicing, and maintaining specialized motor skills [120]. Our results showed that non-musicians resulted benefited from the anodal stimulation, scoring better than the sham group during, in the short-term and in the long-term in the task, while musicians did not resulted benefited from tDCS when compared to their sham group. The better effect on participants with an initial lower performance has different hypothesis. Those purposes are: (1) the type of registration or task [121], (2) the ceiling effect produced as a consequence of a not enough demanding task for over skilled participants [122] and (3) the lower activation of the primary motor cortex, supplementary motor area, premotor cortex and superior parietal lobule [123], indicating a reduced use of primarily motor cortex, as a lower level of brain activity is required [124, 125]. In addition, a study found that non-schizophrenic first-degree relatives of schizophrenia patients had altered MEP response to cathodal tDCS on M1, compared to non-related healthy participants [126]. Assumed the high heritability of schizophrenia, these results enhance the role of genetic variability in the interindividual variability of the response to tDCS.

Therefore, the type of registration task, the baseline performance level, the specific brain areas used for the task, as well as the genetic variability, are facts that need to be taken into account along the application of tDCS. At this point it is important to highlight the need to develop more robust protocols, understanding the individual factors that determine responsiveness.

Other of the principal lacks of tDCS is the extension of the area that results stimulated during its application [117]. Conventional parameters of tDCS may modulate further areas as the specific target placement. For instance, Lang et al. [127] presented a neuroimaging study in which the stimulation of the left primary motor and right frontopolar cortex showed to increase also regional cerebral blood flow (rCBF) on other peripherals and underlying areas.

In response to this demand, a recent improvement of the present technique, namely high definition transcranial direct current stimulation (HD tDCS), has emerged in the field of neuromodulation presenting considerable enhancements. Primarily, HD tDCS electrodes are much smaller than those used by tDCS. Conventional tDCS uses mainly 16–35 cm^2^ electrodes [4] whereas HD tDCS uses ~25 ± 2.5 mm^2^ electrodes provided with a plastic holder, that additionally enhances security. The new configuration of HD tDCS also allows the settlement of more than one anode and cathode, which appears to result in a mayor effect of tDCS and an increase of focality. For instance, the study of Kuo et al. [128] compared a 4 × 1 HD tDCS ring configuration (compounded by four cathodes and one central anode) with a conventional tDCS stimulation of rectangular sponge pads by measuring the motor cortical excitability. In both cases, the current strength was 2 mA and the duration of the stimulation 10 min. Results showed that anodal as well as cathodal tDCS increased or decreased respectively cortico-spinal excitability reaching an effect immediately after the application, while HD-tDCS showed an effect 30 min later. However, the excitability alterations obtained by the conventional mechanism vanished 120 min post tDCS. Interestingly, HD tDCS lasted an effect over the MEP amplitude up to 2 h after the stimulation. Additionally, a better spatial focality using the ring electrode versus conventional rectangular pads was shown by Datta et al. [129].

It is also important to remark that HD tDCS presents the possibility of being applied together with neuroimaging techniques [130], which gives the opportunity to access to detailed information about the cortical activity underlying the stimulation.

However, HD tDCS presents a deficiency compared to conventional tDCS, as it produces a greater scalp sensation during the stimulation, which remains also stable along the stimulation. In this aspect, HD tDCS presents a difficulty for its use on research, as the sham condition traditionally used with tDCS—namely, to administer the stimulation only the first minute—can be difficulty applied without resulting differentiated by the subjects. However, Garnett and Ouden [131] recently validated a sham condition for use in HD tDCS: the anodes and cathodes are located in the same position as in the stimulation condition, but the current direction is changed. The current crosses the scalp in a superficial way, creating in the subject a sensation of stimulation effect, but not reaching cortical areas. Subjects were not able then, to distinguish the stimulation from the sham condition.

Finally, it is important to consider the different effects of HD tDCS in adults and children, as the effect of HD tDCS appears to be stronger in children than in adults. In a study carried on by Minhas et al. [110] comparing the effects HD tDCS on adults and children, the peak electric field of the 4 × 1 high-definition ring configuration was 0.16 and 0.56 V/m (for a disc center to disc center radius of 5 cm), in the adult and child respectively for a 1 mA current stimulation. In addition, modulation of the cortical tissue appeared to extend much deeper (toward the ventricles) at 1.5 mA of current in the child compared to the adult. Therefore, lower parameters have to be applied by using HD tDCS on children.

To sum up, more research about HD tDCS needs to be done in order to define clearly its effects on cortical excitability. However, the fact that it is compatible with the online recording of neuroimaging techniques will allow its study much more easily. On the other hand, it would be desirable to reach less or no-sensation of the stimulation, improving the subject or patient’s comfort and providing the homogeneity between the stimulated and the sham condition. At any rate, HD tDCS is a promising technique that improves the neuromodulation in terms of focality, plasticity and security.

## Conclusions

In the last decade, the advantages of tDCS and its neuromodulation properties have been widely confirmed. In addition, its low side effects, easy management, well-established sham condition and relative low price are crucial keys in order to understand the big attention that tDCS has attracted in the research area in the last years [132].

The number of possible usages of tDCS is enormous, involving the modulation of many behaviors and neurological processes. As seen in the present review, this fact is visible in healthy subjects as well as in patients with diverse neuropathologies, being the neuronal plasticity effect of tDCS its main promising property for its consolidation as a future neurorehabilitation tool. Looking at the most prominent results of tDCS up to date, it is possible to conclude that the most efficient protocols in order to intervent over human motor function, in healthy, as well as in clinical population, involve repetitive sessions of tDCS rather than single sessions, and the intersession time should be at least of 24 h. In addition, these tDCS sessions might report more positive results if they are accompanied by neurorehabilitation, specifically, physical therapy or motor training with the target body part, which ought be performed during the administration of tDCS, if possible, or right after. Regarding the intensity of the stimulation, it should range from 1 to 2 mA, but being not higher as 1 mA in children. Besides the duration of the stimulation of 15 and 30 min has reported positive results, the most used duration time is 20 min. monocephalic anodal tDCS has reported in general, more and better results than bicephalic or cathodal tDCS, and the stimulated areas vary among the corresponding motor cortex area, predominantly, and the cerebellum.

However, the improvement of complementary neuroimaging techniques and scanners with a better neuroanatomical resolution is fundamental in order to augment the knowledge of the basic neuroanatomical/functional brain structures, and consequently, enhance the reliably of the data generated by tDCS [70].

At a microscopic level, it is crucial to keep improving the researching on the biochemical bases that compound the mechanism of action of tDCS, due to the current evidence is still insufficient to set up a complete theory about its modulatory action over the human brain mechanisms [133]. In addition, it is important also to develop further sophisticated methodologies for the analysis of the axon orientation, the dendritic arborization, the role of astrocytes and the electrical field threshold of cortical cells [134].

As seen before, it is crucial to keep on studying the variables responsible for the big variability that tDCS presents. The interaction between the state of brain and the stimulation needs to be studied carefully in order to reach successfully effects of tDCS. In addition, tDCS could be also interesting as a modulator of pharmacological treatments [135].

The advantage of the portability of tDCS has differentiate this device from other neuromodulation techniques, permitting its application together with a variety of motor trainings in different contexes. Moreover, research should focus on the developing of even smaller and light-weighting tDCS equipments, as miniature tDCS devices [136] in order to improve its portability.

Finally, novel devices could supply the traditional tDCS methodology, representing the future of non-invasive neurological interventions. Therefore, as we have seen, HD-tDCS is an evolution of standard tDCS that provides a more focalized, safe and specific stimulation [69, 128].

Besides there is still research to carry on regarding the delimitation of even more effective, safe and long-lasting stimulation parameters, tDCS has demonstrated to be a high promising neuromodulation tool which is able to amplify the improvements seen along the rehabilitation of numerous motor functions.

## Additional files

**Additional file 1: Table S1.** “Effects of tDCS on the motor function in healthy population”, summarizing the most relevant results regarding the application of tDCS on the motor function in healthy participants obtained in the mentioned studies of the present review.

**Additional file 2: Table S2.** “Effects of tDCS on the motor function in clinical population”, summarizing the most relevant results regarding the application of tDCS on the motor function in clinical subjects obtained in the mentioned studies of the present review.
