# Supplementary material for: Transcranial direct current stimulation as a motor neurorehabilitation tool: an empirical review
Source: Biomed Eng Online. 2017 Aug 18;16(Suppl 1):76. doi: 10.1186/s12938-017-0361-8 (PMC5568608; doi:10.1186/s12938-017-0361-8)
Supplement: Supplementary file 1 — Additional file 1: Table S1. “Effects of tDCS on the motor function in healthy population”, summarizing the most relevant results regarding the application of tDCS on the motor function in healthy participants obtained in the mentioned studies of the present review. [file 12938_2017_361_MOESM1_ESM.docx]

# Additional file 1

Table S1. Effects of tDCS on the motor function in healthy population.

| **Effects of tDCS on the motor function in healthy population** | | | | |
| --- | --- | --- | --- | --- |
| **Anodal tDCS** | | | | |
| **Study** | **Subjects** | **Stimulation** | **Schedule** | **Most relevant results** |
| [13] Sriraman et al., 2014 | 12 adults: 8 females, age range 22–32 yr | Monocephalic / atDCS / leg area of right M1 / 1mA | 15 min / 3 sessions (1 session per conditition: - during training, prior training, sham) / ≥7 days of interssesion time | Increased motor performance (ankle dorsiflexion and plantarflexion). Better results with the application of tDCS during the practice rather than prior. Both tDCS groups demonstrated enhanced motor learning when tested 24 h after practice. |
| [64] van Asseldonk et al., 2016 | 10 young adults: 9 male, age range 18–25 yr | Monocephalic and Bicephalic / atDCS / M1 contralateral to the non-preferred leg / 2mA | 10 min /3 sessions (one per conditition: monocephalic, bicephalic, sham) / ≥7 days of interssesion time | Effect over the coordinated motor output and spatiotemporal variables of walking. The tDCS effects were more pronounced when using a bicephalic electrode montage. Large inter-individual variability. |
| [65] Galea et al., 2009 | 16 adults: 10 males, age range 20-45 yr | Monocephalic / atDCS and ctDCS / Right cerebellar cortex, 3cm lateral to the inion / 2 mA | 20 min / 3 sessions (one per condition: atDCS, ctDCS and sham) / ≥16 days of intersession time | Cathodal tDCS decreased and anodal tDCS increased the inhibitory tone the cerebellum exerts over the primary motor cortex, measured by DTI. Cathodal tDCS effects last up to 30 min after the stimulation. |
| [66] Jayaram et al., 2012 | 40 adults: 25 males, age range 20-33 yr | Monocephalic / atDCS and ctDCS / Hemisphere ipsilateral to the fast and slow leg, cerebellum 3 cm lateral to the inion / 2 mA | 15 min / 1 session | Increase or decrease the rate of split-belt treadmill training through anodal or cathodal tDCS, respectively. |
| [68] Madhavan & Stinear et al., 2010 | 10 adults: 7 male, mean age 23 yr | Monocephalic / atDCS / Right and left leg area of M1 / 0.5 mA | 10 min / 2 sessions (one per condition: right and left M1) / Several days of interssesion time | Up-regulation of the target cortex and a down-regulation of the nontarget cortex; and no effects of hemisphere (left, right) or muscle (TA, VL). Significant modulation was evident in 78% of VL and TA muscles. Excitability increased in 60%, but decreased in 18%. For 43% when excitability increased, a simultaneous decrease in excitability was evident in homologous muscle responses. |
| [69] Jeffery et al., 2007 | 8 adults: 6 male, age range 25,3-26 yr | Monocephalic / atDCS and ctDCS / Leg area of the primary motor cortex contralateral to the TA muscle of interest for anodal or cathodal stimulation respectively / 2 mA | 10 min / 3 sessions (each per condition: atDCS, ctDCS and sham) / ≥3 days of intersession time | Increases in the average MEP response measured on the TA muscle of both resting and active states from baseline at 10, 30 and 60 min after the intervention. Resting Resting MEPs following cathodal tDCS remained unchanged except for a small decrease (17%) at the 60-min time point. |
| [121] Saucedo-Márquez et al., 2013 | 30 adults: 15 female, age range 23.14-2.6 yr | Monocephalic / atDCS / Right M1 / 1 mA | 20 min / 3 sessions / 24 h of intersession time | Anodal-tDCS over M1 enhanced learning gains (comprising both online and offline effects) for the SEQTAP task, whereas long-term retention was improved for the FORCE task. |
| [72] Kwon & Kwon, 2013 | 40 adults: 22 male, age range 22.97-2.21 yr | Monocephalic / atDCS / M1 and pre-SMA / 1 mA | 10 min / 3 sessions (one per condition: preSMA tDCS, M1 tDCS and sham) / ≥24 h of intersession time | Significant enhancement of the stop movement of the non-dominant hand during performance of the SST after delivery of anodal tDCS of the pre-SMA, but not under the sham condition. |
| **Cathodal tDCS** | | | | |
| **Study** | **Subjects** | **Stimulation** | **Schedule** | **Most relevant results** |
| [65] Galea et al., 2009 | 16 adults: 10 males, age range 20-45 yr | Monocephalic / atDCS and ctDCS / Right cerebellar cortex, 3cm lateral to the inion / 2 mA | 20 min / 3 sessions (one per condition: atDCS, ctDCS and sham) / ≥16 days of intersession time | Cathodal tDCS decreased and anodal tDCS increased the inhibitory tone the cerebellum exerts over the primary motor cortex, measured by DTI. Cathodal tDCS effects last up to 30 min after the stimulation. |
| [66] Jayaram et al., 2012 | 40 adults: 25 males, age range 20-33 yr | Monocephalic / atDCS and ctDCS / Hemisphere ipsilateral to the fast and slow leg, cerebellum 3 cm lateral to the inion / 2 mA | 15 min / 1 session | Increase or decrease the rate of split-belt treadmill training through anodal or cathodal tDCS, respectively. |
| [69] Jeffery et al., 2007 | 8 adults: 6 male, age range 25,3-26 yr | Monocephalic / atDCS and ctDCS / Leg area of the primary motor cortex contralateral to the TA muscle of interest for anodal or cathodal stimulation respectively / 2 mA | 10 min / 3 sessions (each per condition: atDCS, ctDCS and sham) / ≥3 days of intersession time | Increases in the average MEP response measured on the TA muscle of both resting and active states from baseline at 10, 30 and 60 min after the intervention. Resting Resting MEPs following cathodal tDCS remained unchanged except for a small decrease (17%) at the 60-min time point. |

Table S1 shows the most relevant results regarding the application of tDCS on the motor function in healthy participants obtained in the mentioned studies of the present review. From left to right, we find in the first column, the reference number used along the text and the authors and year or the study. The second column descripts the type of subjects: number of participants, if children/adolescents/young adults, gender and age range. The third column defines the kind of stimulation: bicephalic when the reference electrode is located in the contralateral area and manocephalic when the cathode is located somewhere else, type of active electrode (atDCS, ctDCS…), location of the active electrode and the intensity (in mA). The fourth column resumes the schedule of the study: minutes of stimulation, number of sessions and intersession-time. Finally, the last column defines the most relevant results obtained in the stimulation of tDCS regarding the motor function in healthy humans. The table is divided into two parts (Anodal tDCS and Cathodal tDCS) depending on the use of Anodal electrode as active electrode in the first case, or also the Cathodal electrode as active electrode, in the second case. Other results not related to the topic of the present review will be omitted. Please consult the literature references for the complete information of the study.
