# Supplementary material for: Transcranial direct current stimulation as a motor neurorehabilitation tool: an empirical review
Source: Biomed Eng Online. 2017 Aug 18;16(Suppl 1):76. doi: 10.1186/s12938-017-0361-8 (PMC5568608; doi:10.1186/s12938-017-0361-8)
Supplement: Supplementary file 2 — Additional file 2: Table S2. “Effects of tDCS on the motor function in clinical population”, summarizing the most relevant results regarding the application of tDCS on the motor function in clinical subjects obtained in the mentioned studies of the present review. [file 12938_2017_361_MOESM2_ESM.docx]

# Additional file 2

Table S2. Effects of tDCS on the motor function in clinical population.

| **Effects of tDCS on the motor function in clinical population** | | | | | |
| --- | --- | --- | --- | --- | --- |
| **Stroke and tDCS** | | | | | |
| **Study** | **Subjects** | **Clinical profile** | **Stimulation** | **Schedule** | **Most relevant results** |
| [75] Nair et al., 2011 | 14 adults: 9 male, mean age 55.8. First unic unihemispheric stroke. | 6 patients with lesion in right hemisphere; 8 in left hemisphere. Time elapsed after stroke: 33 ± 20 for cathodal group; 28 ± 28 for sham group. | Monocephalic / ctDCS / Contralesional motor region C3 or C4 / 1 mA | 30 min of tDCS at the beginning of a 60 min Occupational Therapy / 5 sessions / 25 h of intersession time | Positive effects on Range-Of-Motion 5 and 7 days after the intervention and Upper-Extremity Fugl-Meyer Assessment scores 7 days after the intervention. |
| [31] Lindenberg et al., 2010 | 20 adults: 15 male, mean age 58,75 yr. | Chronic stroke patients. First unic ischemic stroke in the territory of the medial cerebral artery at least 5 months prior to enrollment. | Bicephalic / atDCS and ctDCS / Anode placed over the ipsilesional and the cathode over the contralesional motor cortex (C3 and C4) / 1.5 mA | 30 min / 1 session | Better results in the Upper Extremity Fugl-Meyer and Wolf Motor Function Test after the bicephalic intervention compared to sham condition. Effects persisted for at least 1 week after the intervention. |
| [77] Hummel et al., 2005 | 6 adults: 4 male, age range 38–84 yr. | Patients with a history of a single ischaemic subcortical stroke cerebral leading to initial severe upper arm motor paresis (MRC grade <2). | Monocephalic / atDCS / M1 of affected hemisphere / 1 mA | 20 min / 1 session | Functional improvement in the paretic hand measured by the Jebsen-Taylor Hand Function test that outlasted the stimulation period and was present in every patient tested. |
| [78] Rosso et al., 2014 | 25 adults: 13 female, age range 18-85 yr. | First stroke in the left middle cerebral artery territory. Presence of aphasia based on item 9 of the National Institute of Health stroke scale. | Monocephalic / ctDCS / Stimulation electrode was placed using a neuronavigator (Nexstim Eximia NBS, v. 3.2.1) with the electrode center placed on the ascendant ramus of the lateral sulcus separating the pars triangularis and pars opercularis of the inferior frontal gyrus, which were located using the anatomical images from each participant / 1 mA | 15 min / 2 sessions (ctDCS and sham) / 2 h of intersession time | Picture naming accuracy was improved in the group with Broca’s area damage, whereas no changes or worsening were observed in the intact Broca’s area group. |
| **Dysphagia and tDCS** | | | | | |
| **Study** | **Subjects** | **Clinical profile** | **Stimulation** | **Schedule** | **Most relevant results** |
| [85] Kumar et al., 2011 | 14 adults: 7 female, mean age 74,85 yr. | First ischemic stroke and dysphagia secondary to a new unilateral hemispheric infarction. | Monocephalic / atDCS / Over the undamaged hemisphere, mid-distance between C3 and T3 on the left or C4 and T4 on the right / 2 mA | 30 min / 5 sessions / 24 h of intersession time | Improvements on the Outcome and Severity Scale score |
| [86] Yang et al., 2012 | 16 adults. | Post-stroke dysphagia. | Monocephalic / atDCS / Over the pharyngeal motor cortex of the affected hemisphere / 1 mA | 20 min / 10 sessions / ≥24 h of intersession time | Effects of Functional Dysphagia scale using video fluoroscopic swallowing measure three months after the intervention. |
| **Parkinson’s disease and tDCS** | | | | | |
| **Study** | **Subjects** | **Clinical profile** | **Stimulation** | **Schedule** | **Most relevant results** |
| [89] Fregni et al., 2006 | 17 adults: 11 male, age range 45-79. | Post-stroke patients with idiopathic Parkinson’s Disease who fulfilled the U.K. Parkinson’s Disease Brain Bank criteria. | Monocephalic / atDCS and ctDCS / Left M1 / 1 mA | 20 min / 2 sessions (each per condition – atDCS/ctDCS and sham) / ≥48 h of intersession time | atDCS increased and ctDCS decreased MEPs. Improvement of the motor function in Unified Parkinson’s Disease Rating Scale after atDCS. |
| [90] Valentino et al., 2014 | 10 adults: 5 female, mean age 72,3. | Patients with Parkinson’s Disease in a Hoehn and Yahr stage of 2 to 4 while “off” medication, scoring 3 or more on item 3 of the Freezing of Gait Questionnaire (FOG-Q). FOG persisting in the “on” state. | Monocephalic / atDCS / M1 corresponding to the leg with which the patient usually started walking after a FOG episode / 2 mA | 20 min / 5 sessions / 24 h of intersession time | Decrease of the number, and duration of FOGS, decrease in the number of steps and time needed to complete test. Results assessed by the Stand Walk Sit (SWS) test during ON-state. |
| [91] Ferruchi et al., 2016 | 9 adults: 5 male, age range 60-85. | Idiopathic PD patients with levodopa-induced dyskinesias (LIDs) (Hoehn & Yahr scale score 2–3; illness duration 7–16; Mini Mental State Examination score 26–30). | Bicephalic / atDCS / M1 and Cerebellar tDCS / 2 mA | 20 min / 5 sessions / 24 h of intersession time | After 5 days, bilateral anodal cerebellar tDCS and M1 tDCS reduced LIDs in patients with PD assessed by the UPDRS IV for dykinesias. |
| [^21^] Benninger et al., 2010 | 25 adults: 16 male, age range 40-77. | PD patients according to UK PD Brain Bank criteria in a Hoehn and Yahr (HY) stage of 2 to 4 while “off” medication. Patients had to have slowing of gait defined as a time of 6 seconds or more to walk 10 meters. | Monocephalic / atDCS / M1 and PFC / 2 mA | 20 min / 8 sessions / 48 h of intersession time | tDCS improved gait by some measures for a short time and improved bradykinesia in both the on and off states for longer than 3 months. Changes in UPDRS, reaction time, physical and mental well being, and self-assessed mobility did not differ between the tDCS and sham interventions. |
| **Multiple sclerosis/Amyotrophic lateral sclerosis and tDCS** | | | | | |
| **Study** | **Subjects** | **Clinical profil** | **Stimulation** | **Schedule** | **Most relevant results** |
| [94] Cuypers et al., 2013 | 10 adults: 6 female, age range 27-65. | Stable Multiple sclerosis patients (no relapse 3 months prior to inclusion). | Monocephalic / atDCS / M1 contralateral to the more impaired hand / 1 mA | 20 min / 2 sessions (each per condition: atDCS or sham) / ≥ one week of intersession time | Increased corticospinal excitability output and projection strength after atDCS. |
| [95] Meesen et al., 2014 | 31 adults: 22 female, age range 27-65. | Multiple Sclerosis patients showing no relapse for at least 3 months prior to the study. | Monocephalic / atDCS / M1 contralateral to the more impaired hand 1 mA | 20 min / 1 session | No changes in the unimanual motor sequence-training task score. |
| [^96^] Mori et al., 2013 | 20 adults: 12 female, age range 25-61. | Relapsing remitting Multiple Sclerosis with diminished tactile perception at the upper limb | Monocephalic / atDCS / Contralateral S1 / 2 mA | 20 min / 5 sessions / 24 h of intersession time | Better performing in the Grating Orientation task observable at day 5 and up to 2 weeks after the intervention. |
| [^97^] Mori et al., 2010 | 19 aduls: 11 female, mean age 44.8. | Relapsing remitting Multiple Sclerosis in remitting phase, presenting with chronic, drug-resistant, neuropathic pain. | Monocephalic / atDCS / Contralateral M1 / 2 mA | 20 min / 5 sessions / 24 h of intersession time | Improvements in VAS for pain scores from day 3 up to week 4. Improvements also in McGill and Multiple Sclerosis Quality of Life-54 questionnaire from 1 up to 4 weeks after the treatment. No effects seen on VAS for anxiety and Beck Depression Inventory. |
| [^99^] Munneke et al., 2011 | 20 adults: gender and age not reported. | 10 patients with sporadic, categorized as having clinically probable Amyotrophic lateral sclerosis according to the revised El Escorial criteria. | Monocephalic / ctDCS / Contralateral M1 / 1 mA | 7, 11, or 15 minutes / 3 sessions (each per condition: time of stimulation) / 1 week of intersession time | No cortical excitability variations after any of the stimulation conditions on Amyotrophic lateral sclerosis, but healthy controls showed a decrease of cortical excitability with lengthening of the stimulation duration. |
| [^100^] Quartarone et al., 2007 | 16 adults: 8 sporadic | Amyotrophic lateral sclerosis patients according to the revised El Escorial criteria: 4 female, age range 43-68. | Monocephalic / atDCS and ctDCS / Contralateral right FDI muscle representation in the motor cortex / 1 mA | 7 min / 2 sessions (each per condition: atDCS or ctDCS) / ≈1 week of intersession time | Sustained excitability changes in healthy subjects of about, with anodal tDCS inducing facilitation and cathodal tDCS leading to inhibition. The excitability of M1 of ALS patients was not affected by anodal or cathodal tDCS. |
| **Spinal cord injury and tDCS** | | | | | |
| **Study** | **Subjects** | **Clinical profil** | **Stimulation** | **Schedule** | **Most relevant results** |
| [^102^] Silva et al., 2013 | 1 adult: male, 25 years. | Patient with total chronic Spinal Cord Injury (SCI) (7 years), between T11 and T12 levels, not spastic, sedentary and right-handed. | Bicephalic / atDCS / Both M1 -Cz (central zero) at 4.5 cm on the left hemisphere and 4.5 cm on the right hemisphere- / 2 mA | 13 min / 3 sessions (each per condition: atDCS, Sham and control) / 48 h of intersession time. | General improvement in in exercise time and power, perceived exertion, glucose levels, and the time needed to reach the heart rate threshold. |
| [^103^] Murray et al., 2015 | 9 adults. | Chronic SCI and motor dysfunction in wrist extensor muscles. | Monocephalic / atDCS / Left M1 (extensor carpi radialis muscle representation) / 1 mA and 2 mA | 20 min / 3 sessions (1 per condition: atDCS at 1 mA, atDCS at 2 mA and sham) / ≥1 week of intersession time. | atDCS transiently raised corticospinal excitability to affected muscles in patients with chronic SCI after 2mA stimulation. Sensory perception improved with both 1 and 2mA stimulation. |
| [^104^] Hubli et al., 2013 | 34 adults. | 17 motor complete SCI patients: male, mean age 35.9. The levels of lesion ranged from C3 to T6 and lesion duration was 96.0 months. | Anodal Transcutaneous spinal direct current stimulation (tsDCS) and cathode on the left shoulder / Between the spinous processes T11 and T12 / 2.5 mA | 20 min / 4 sessions (each per condition: anodal, cathodal, sham tsDCS or locomotion) / ≥1 week of intersession time. | Specific differences in spinal reflex behavior, where patients showed higher changes in spinal reflex amplitude after a-tsDCS than healthy subjects, exhibiting even better results than receiving a session of assisted walking in the driven gait orthosis "Lokomat". |
| **Restless legs syndrome and tDCS** | | | | | |
| **Study** | **Subjects** | **Clinical profil** | **Stimulation** | **Schedule** | **Most relevant results** |
| [^107^] Heide et al., 2014 | 34 adults. | 20 patients: mean age: 56.2, 15 female and 5 male with primary idiopathic RLS. International Restless Legs Severity Scale (IRLSS) score: 27. | Anodal and Cathodal Transcutaneous spinal direct current stimulation (tsDCS) / Over the thoracic spinal cord about 2 cm left paravertebrally and longitudinally to the Th11 level, reference electrode was positioned over the right supraclavicular region / 2.5 mA | 15 min / 3 sessions (each per condition: anodal, cathodal and sham) / ≥1 week of intersession time. | Application of anodal stimulation led to a decreased H2/H1-ratio for 0.2 and 0.3 s interstimulus intervals in patients. Anodal and cathodal stimulation led to a reduction in restless legs symptoms on the VAS. |
| [^108^] Koo et al., 2015 | 33 adults: female, age range 18–70 yr. | With a diagnosis of idiopathic RLS. (1) Duration of RLS ≥1 year; (2) having symptoms ≥3 times per week; and (3) IRLS score ≥20, indicating severe symptomatology. | Bicephalic / atDCS and ctDCS/ Leg area of M1 / 2 mA | 20 min / 15 sessions (5 consecutive sessions (from monday to friday) per condition: anodal, cathodal and sham) during 3 weeks. | No differences among the groups in the IRLS scores, as well as the responder rate in the CGI-I. |
| **Cerebral palsy in children and tDCS** | | | | | |
| **Study** | **Subjects** | **Clinical profil** | **Stimulation** | **Schedule** | **Most relevant results** |
| [^111^] de Almeida et al., 2015 | 24 children: age range: 5-12 yr. | Diagnosis of spastic CP; classification on levels I, II or III of the Gross Motor Function Classification System (GMFCS); independent gait for at least 12 months. | Monocephalic / atDCS / Non-dominant hemisphere M1 / 1 mA | 20 min / 10 sessions of atDCS and treadmill training during 2 weeks. | atDCS group exhibited better results in comparison to the control group on anteroposterior sway (eyes open and closed, mediolateral sway (eyes closed) and the Pediatric Balance Scale both one week and one month after the completion of the protocol. |
| [^113^] Aree-Uea et al., 2014 | 46 children and adolescents: age range 8-18 yr. | Diagnosis of spastic CP based on standard diagnostic criteria, CP with gross motor function classification system (GMFCS) levels II-IV, upper limb spasticity grade 1 to 3, based on the modified Ashworth Scale. | Monocephalic / atDCS / Left M1 or C3 locus / 1 mA | 20 min / 5 sessions on 5 consecutive days. | atDCS evidenced significantly more pre- to immediately post-treatment reductions in spasticity than participants assigned to the sham (for shoulder, wrist, and fingers respectively). Improvement in spasticity maintained for at least 48 hours for wrist joints. |
| [^114^] Lazzari et al., 2015 | 20 children: age range: 4-12 yr. | Patients with CP, presenting levels I, II or III of the Gross Motor Function Classification System (GMFCS); independent gait for at least 12 months. | Monocephalic / atDCS / M1 / 1 mA | 20 min of atDCS with mobility training on virtual reality / 1 session | Increase in the body sway velocity measured by the force plate. |
| [^115^] Grecco et al., 2015 | 20 children: age range 5-10 yr. | Diagnosis of spastic diparetic cerebral palsy, classification on levels II or III of the Gross Motor Function Classification System, independent gait for at least 12 months. | Monocephalic / atDCS / M1 contralateral to the lower limb with greater motor impairment / 1 mA | 20 min / atDCS with gait training on virtual reality / 10 sessions on 2 weeks. | Improvements in gait velocity, cadence, gross motor function and independent mobility. |

Table S2 shows the most relevant results regarding the application of tDCS on the motor function in clinical subjects obtained in the mentioned studies of the present review. From left to right, we find in the first column, the reference number used along the text and the authors and year or the study. The second column describes the type of subjects: number of participants, if children/adolescents/young adults, gender and age range. The third column describes the clinical profile of the participants. The fourth column defines the kind of stimulation: bicephalic when the reference electrode is located in the contralateral area and manocephalic when the cathode is located somewhere else, type of active electrode (atDCS, ctDCS…), location of the active electrode and the intensity (in mA). The fifth column resumes the schedule of the study: minutes of stimulation, number of sessions and intersession-time, or duration of the treatment. Finally, the sixth column defines the most relevant clinical results obtained in the stimulation of tDCS after the treatment. The table is divided depending on the pathology treated, from top to bottom, in the same order as seen in the present manuscript. Other results not related to the topic of the present review will be omitted. Please consult the literature references for the complete information of the study.
